# Regulation of Circadian and Acute Activity Levels by the Murine Suprachiasmatic Nuclei

**DOI:** 10.1371/journal.pone.0110172

**Published:** 2014-10-08

**Authors:** Thijs Houben, Claudia P. Coomans, Johanna H. Meijer

**Affiliations:** Department of Molecular Cell Biology, Laboratory for Neurophysiology, Leiden University Medical Center, Leiden, the Netherlands; University of Lübeck, Germany

## Abstract

The suprachiasmatic nuclei (SCN) coordinate the daily sleep-wake cycle by generating a circadian rhythm in electrical impulse frequency. While period and phase of the SCN rhythm have been considered as major output parameters, we propose that the waveform of the rhythm of the SCN also has significance. Using implanted micro-electrodes, we recorded SCN impulse frequency in freely moving mice and manipulated its circadian waveform by exposing mice to light-dark (LD) cycle durations ranging from 22 hours (LD 11∶11) to 26 hours (LD 13∶13). Adaptation to long T-cycles (>24 h) resulted in a trough in electrical activity at the beginning of the night while in short T-cycles (<24 h), SCN activity reached a trough at the end of night. In all T-cycle durations, the intensity of behavioral activity was maximal during the trough of SCN electrical activity and correlated negatively with increasing levels of SCN activity. Interestingly, small changes in T-cycle duration could induce large changes in waveform and in the time of trough (about 3.5 h), and accordingly in the timing of behavioral activity. At a smaller timescale (minutes to hours), we observed a negative correlation between SCN activity and behavioral activity, and acute silencing of SCN neurons by tetrodotoxin (TTX) during the inactive phase of the animal triggered behavioral activity. Thus, the SCN electrical activity levels appear crucially involved in determining the temporal profile of behavioral activity and controls behavior beyond the circadian time domain.

## Introduction

Twenty four hour rhythms in physiology and behavior are ubiquitous in the animal kingdom, and are an evolutionary adaptation to the environmental rhythms caused by the rotation of the earth. In mammals, the hypothalamic suprachiasmatic nuclei (SCN) function as a master clock that coordinate a multitude of rhythmic processes in the body. The rhythms of the SCN are explained by the interaction between an intra-neuronal molecular network involving circadian genes and their products and intercellular communication within the SCN neuronal network [1], [2]. The SCN is synchronized to the external light-dark cycle via a direct input from the retina. While the intracellular circadian molecular network is rhythmic in many peripheral tissues, the SCN is necessary for coordinating these rhythms and for driving the 24 hour rhythm in behavioral activity levels and sleep [3]–[6]. Several output factors of the SCN are triggered and released by electrical activity of SCN neurons, including arginine vasopressin, gamma-amino butyric acid, glutamate, prokineticin, cardiotrophin-like cytokine and vasoactive intestinal peptide [7]–[12].

Although there is strong evidence that the SCN regulates the period and phase of behavioral activity patterns [5], [13], it is at present not known if the SCN also plays a role in regulating the level of behavioral activity. On the one hand, the presence of molecular peripheral oscillators and their persistence after SCN ablation is interpreted as evidence that the rhythmic output of the SCN acts merely as synchronizer of peripheral oscillators [14], [15]. On the other hand, the SCN may have a more extensive role and its electrical activity waveform may have implications for the temporal distribution of behavior. We therefore investigated whether the waveform of the SCN is significant for the regulation of the pattern of behavioral activity, or whether alternatively the SCN signal is merely setting a gate for behavioral activity during the night. This question is of immediate importance for circumstances where disturbances in rhythms have been observed, such as in aging or in neurodegenerative disorders [16], [17]. To explore the role of the SCN electrical activity waveform, we measured multi-unit activity (MUA) in the SCN of freely moving animals using implanted micro-electrodes. We found that we could modify the SCN electrical activity waveform by gradually lengthening or shortening the duration of the external T-cycle and used this finding to investigate the influence of the electrical activity levels on the temporal profile of behavioral activity. We found that the timing and intensity of behavioral activity was completely predictable by the trough in SCN activity. Directly suppressing SCN electrical activity by SCN injections of tetrodotoxin (TTX) during the subjective day stimulated behavioral activity. Together, these results demonstrate that the waveform of the SCN electrical activity rhythm determines the temporal pattern of behavioral activity to a large extent.

## Materials and Methods

### Ethics Statement

All animals were handled in accordance to Dutch law using a protocol approved by the Animal Ethics Committee from the Leiden University Medical Center (Leiden, the Netherlands).

### Animals and Housing

Male C57Bl/6JOlaHsd mice (Harlan, Horst, the Netherlands) were obtained at an age of 8 weeks and housed individually. The cages had a floor surface of 22 cm×38 cm and were equipped with a running wheel with a diameter of 24.35 cm. Sensors on the running wheel were connected to a ClockLab data collection system (Actimetrics, IL, USA) that recorded the number of wheel revolutions in 1 minute bins. All recordings were performed in light-tight ventilated cabinets in a climate-controlled room (21°C; 40–50% humidity). Food (chow, RM3; Special Diet Services, Sussex, U.K.) and tap water were available *ad libitum* during the entire experiment.

### T-Cycles

Inside the light-tight ventilated cabinets, experimental light-dark cycles (T-cycles) were provided using computer controlled broad-spectrum fluorescent tubes. Upon arrival, animals were exposed to a T-cycle of 12 hours light and 12 hours of darkness (LD 12∶12, T = 24 h). After a period of at least 14 days in LD 12∶12, half of the animals were exposed to a series of shortening T-cycles in which the cycle length was decreased by 20 minutes every 7th cycle. This decrease was applied by shortening the dark as well as the light part of the cycle by 10 minutes. The other group of animals was exposed to a series of lengthening T-cycles in which the cycle length was increased by 20 minutes every 7th cycle. This increase was applied by lengthening the dark as well as the light part of the cycle by 10 minutes. For comparison between different T-cycle durations, all timepoints were expressed as Zeitgeber time (ZT). ZT divides the entraining T-cycle into 24 equal time units, with ZT = 0 defined as the start of the light period. ZT is calculated by dividing the hours that have passed since the start of the last light period by the duration of the T-cycle and multiplying the result by 24.

### Electrode Implantations

Under deep anesthesia (ketamine, 100 mg/kg; xylazine, 10 mg/kg and atropine, 0.1 mg/kg), animals were implanted with tripolar stainless steel electrodes (125 µM, Plastics One, Roanoke, Virginia, USA), under a 5° angle in the coronal plane. Two of the electrodes were insulated and twisted together with the exposed recording tips aimed at the SCN. The following coordinates were used from Bregma: 0.46 mm anterior, 0.61 mm lateral and 5.38 mm ventral [18]. The third electrode was placed in the cortex and used as ground. Post-operative pain relief was provided by a subcutaneous injection of buprenorphine (0.05 mg/kg). After surgery, animals were allowed to recover in their homecage for 7 days.

### SCN Electrical Activity Recordings

For recording of SCN electrical activity, animals were placed in a recording chamber with ad libitum availability of water and food. The implanted electrodes were connected to a data-acquisition system using a counterbalanced, low-torque electrical swivel to minimize strain on the electrode. Electrical activity was amplified using an in-house developed differential amplifier and spikes were counted using amplitude-based spike triggers. Additional sensors in the recording setup recorded behavioral activity (passive infrared motion detection sensor, PIR) and light levels (light-dependent resistor) with a ten second time resolution. Recordings were obtained under different T-cycle durations ranging from T = 22 h up to T = 26 h. At the end of the recordings, animals were killed and electrodes were marked by passing an electrical current through the electrode for histological verification of the electrode location.

### Analysis of Electrophysiological and Behavioral Data

**Figure 1:**
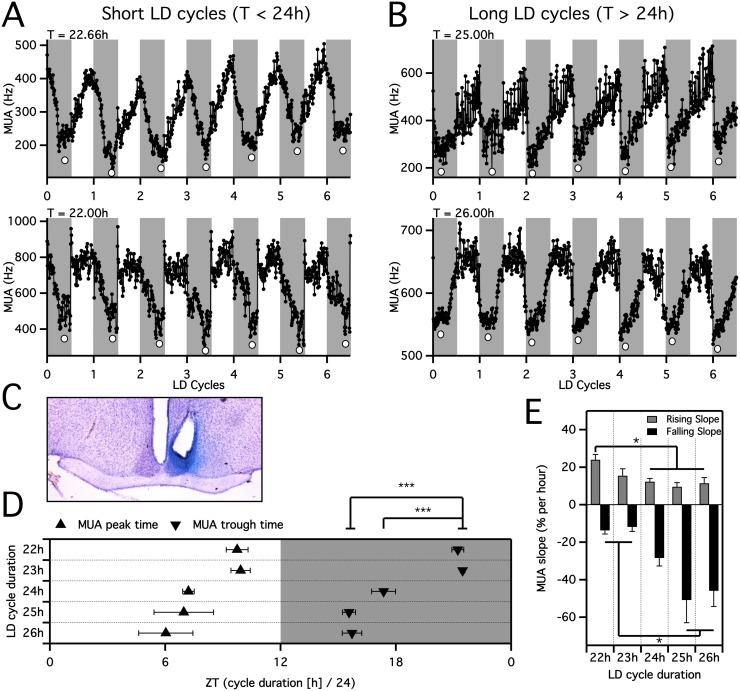
The waveform of SCN electrical activity is modulated by adaptation to short or long T-cycles. A. Raw electrical activity recordings from 2 individual animals adapted to short T-cycles (top: T = 22.66 h, bottom: T = 22 h, 10 minute time-resolution) over 6 cycles. Grey background indicates lights off. Round markers below the electrical activity trace indicate the timing of the trough. B. Recordings of SCN electrical activity during long T-cycles (top: T = 25 h, bottom: T = 26 h). Note the timing of the troughs at the beginning of the nights. C. Representative histological section confirming placement of electrode in the suprachiasmatic nucleus. D. The SCN electrical activity trough occurred late in the night during short T-cycles, near the middle of the night during T = 24 h and in the early night during long T-cycles. (***P<0.001). Peak SCN firing rates were near the end of the light period in T = 22 h and T = 23 h and near the middle of the light period in T = 24 h, T = 25 h and T = 26 h. E. Significant differences in the steepness of the declining and rising SCN electrical activity slopes (*P<0.05).

Offline, raw data files from individual recordings were imported into Igor Pro (version 6.2, Wavemetrics, Inc,. USA) and averaged with a 10 minute time resolution by calculating the average SCN electrical activity level (Hz) and the fraction of 10 second bins in which behavioral activity was detected. For each T-cycle duration, stable recordings of at least 5 days were used to calculate the average SCN electrical activity and behavioral pattern. A few animals did not entrain to T-cycles shorter than T = 22.66 h, however, the data were included only when the animals were still entrained to the T-cycles, so T = 23 h. For quantification of the SCN electrical activity waveform, the averaged pattern was normalized by setting the trough to 0% and the peak to 100%. In some of the T = 22 h and T = 23 h recordings, a transient light response was present at the onset of the light period which was excluded from analysis as it exceeded the height of the circadian activity peak (e.g. Fig. 1A, bottom left). No other data were excluded from the analyses. The waveform was characterized by determining the time points at which SCN electrical activity reached levels of 10%, 25%, 50%, 75% and 90%. Trough time was defined as the median time point between 10% electrical activity on the falling and rising slopes. Peak time was defined as the median time point between 90% SCN electrical activity on the rising and falling slopes. The steepness of the rising and falling slopes was calculated from the time difference between 25% and 75% SCN electrical activity level on the rising and falling slopes. For each recording, the corresponding averaged behavioral activity pattern was used to determine the start and end of the active phase. The median of behavioral activity was determined by first calculating the cumulative amount of activity as a function of time for one T-cycle, starting at the middle of the light period. The median was then defined as the time when the cumulative activity exceeded half of the total amount of activity per T-cycle. The relationship between the level of SCN electrical activity and behavioral activity was calculated by dividing SCN electrical activity in 10% bins from 0% to 100% and calculating the percentage of total behavioral activity for each bin. The fraction of time during which electrical activity was at each particular level was determined by dividing the number of 10 minute bins in which SCN activity was at this level by the total number of 10 minute bins within one T-cycle. The relationship between the SCN electrical activity level and the intensity of behavioral activity was calculated at a circadian and ultradian domain (i.e. time scale <24 h).

### Analysis of Running Wheel Recordings

Behavioral activity profiles were constructed for short and long T-cycle durations using the ClockLab (Actimetrics, Il, USA) analysis module and exported for analysis in Igor Pro (version 6.2, Wavemetrics, Inc., USA).

### Suppressing SCN Electrical Activity by local TTX injections

Animals were implanted with a guide cannula attached to electrodes in the SCN as described above (MS333-3, PlasticsOne, Roanoke, Virginia, USA). After recovery, animals were placed in a recording chamber on LD 12∶12. The implanted electrodes were connected as described above. The mice were handled for at least 4 consecutive days. To avoid masking effects of light on behavior (Mrosovsky N et al, 1999), lights were off during the entire day of the SCN injection. The animals received the injection at circadian time (CT) 6 and were injected with either 0.2 µl TTX (1 µM in artificial cerebrospinal fluid (aCSF)) or vehicle directly into the SCN via the implanted guide cannula. TTX at this concentration reversibly inhibits neuronal activity by blocking the voltage-gated fast sodium channels, thereby inhibiting the generation of action potentials [19].

### Statistical Analysis

Differences in the phase of SCN electrical activity and/or behavioral activity between different T-cycle durations were calculated with one-way ANOVAs followed by post-hoc Bonferroni corrected t-tests. The timing of SCN electrical activity troughs was compared to the median behavioral activity time using paired sample t-tests, followed by Bonferroni correction for the number of comparisons. The relationship between SCN electrical activity levels and the intensity of behavioral activity was quantified by linear regression for SCN activity levels between 0 and 50% and compared between different T-cycle durations using an F-test. For all tests, *P*<0.05 was considered to be significant.

## Results

### T-cycle Duration Affects the Time of the SCN Electrical Activity Waveform

SCN electrical impulse frequencies were successfully recorded in animals that were entrained to light-dark (LD) cycles of 24 hours (T = 24 h), to shorter T-cycles with a minimum of 22 hours (T = 22 h), and to longer T-cycles with a maximum of 26 hours (T = 26 h, Figs. 1A and B). Under T = 24 h, low electrical activity levels were reached during the dark part of the LD cycle (trough at ZT 17.36 h+/−0.62), and high electrical activity occurred during the light part of the LD cycle (peak at ZT 7.21+/−0.30). Histological verification of the recording site confirmed that recordings were from the SCN (Fig. 1C). The transitions between peak and trough of the electrical activity occurred during the transitions between light and darkness. The SCN electrical activity rhythms of all animals recorded during T-cycles of T = 23 h (n = 4), T = 24 h (n = 6), T = 25 h (n = 4) and T = 26 h (n = 5) adapted unfailingly. However, the SCN electrical activity rhythms of animals exposed to T-cycle durations shorter than T = 23 h had more difficulty accommodating. Three animals failed to entrain to shortening the T-cycle from T = 22.66 h to T = 22.33 h and two animals lost entrainment upon shortening the T-cycle from T = 22.33 h to T = 22 h. Four animals adjusted their SCN electrical activity rhythms successfully to T = 22 h. The time of the trough of the SCN electrical activity rhythm differed among the T-cycle durations (Fig. 1D, *P*<0.0001, one-way ANOVA). It was at the end of the dark period during short T-cycles, at the middle of the dark period during T = 24 h and at the beginning of the dark period during long T-cycles (Fig. 1D). The time of the trough differed significantly between short, long, and 24 hour T-cycles, but did not differ significantly between T = 25 h and T = 26 h or between T = 23 h and T = 22 h. The peak in the SCN rhythm was at the end of the light period during short T-cycles and at the middle of the light period when the T-cycle comprised 24 or more hours. One way ANOVA revealed a significant variance of peak time among different T-cycles (*P* = 0.0227).

### T-cycle Duration Modifies the Slope of the SCN Electrical Activity Waveform

The different T-cycle durations led to significant differences in the steepness of the falling and rising slopes of the SCN electrical activity rhythm. During short T-cycles, the rate of change during the declining slope was low (T = 22 h: 13.68% +/−1.96% per hour, T = 23 h: 11.81% +/−2.42% per hour), in T = 24 h it was intermediate (28.40% +/−4.30% per hour) and under long T-cycles it was high (T = 25 h: 50.82% +/−12.15% per hour, T = 26 h: 45.90% +/−8.33% per hour). The rate of change during the rising slope was highest in T = 22 h (24.05% per hour), and significantly different from T = 24 h, T = 25 h and T = 26 h (*P*<0.05, Fig. 1E, Table 1).

**Table 1:** Relationship between SCN electrical activity levels and behavioral activity timing and intensity under short, intermediate, or long T-cycles.

Variable	*P* value	T = 22 h	T = 23 h	T = 24 h	T = 25 h	T = 26 h
*Timing of electrical* *activity and behavior:*						
Behavior median (ZT)	<0.0001	20.67+/−0.26	20.96+/−0.26	17.94+/−0.29	16.72+/−0.34	16.06+/−0.54
SCN MUA trough (ZT)	<0.0001	21.22+/−0.31	21.47+/−0.12	17.36+/−0.62	15.56+/−0.34	15.72+/−0.51
Paired samples T-tests(*P* value)		0.2071	0.2605	0.2664	0.0154	0.4217
*Behavioral intensity vs* *SCN MUA level:*						
Linear regression slope(f(y))	0.4736	f(y) = −0.0018x	f(y) = −0.0018x	f(y) = −0.0018x	f(y) = −0.0016x	f(y) = −0.0022x
Y intercept (y(0))	1.0000	y(0) = 0.1898	y(0) = 0.1885	y(0) = 0.1923	y(0) = 0.1792	y(0) = 0.2088
Goodness of fit (R^2^)		0.75	0.57	0.68	0.49	0.52
N		6	4	6	4	5

The relationship between SCN electrical activity levels and behavioral activity timing and intensity is unaltered by exposure to short or long T-cycles. The timing of the SCN electrical activity trough and the median of behavioral activity are similarly and significantly affected by the duration of the T-cycle (*P*-values for one-way ANOVA). In all T-cycle durations, the timing of electrical activity trough and behavior median was equal (Bonferroni corrected paired samples t-tests). The interaction between the intensity of behavioral activity and the level of SCN electrical activity was quantified by linear regression from 0% to 50% SCN electrical activity level. Slopes and elevations of the regression lines were equal under all T-cycle durations (*P*-values for F-Test).

### T-cycle Duration Determines the Distribution of Behavioral Activity

**Figure 2:**
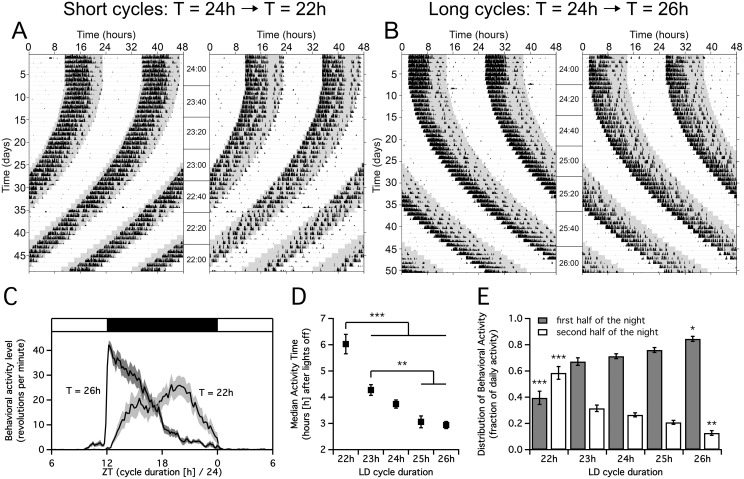
Changes in timing and waveform of behavioral activity during short and long T-cycle durations. A, B. Actograms display running wheel activity of individual mice exposed to gradually shortening (A) or gradually lengthening (B) T-cycles. Data are double plotted. Background color indicates lights on (white) or lights off (grey). Note that as T-cycles progressively shorten, behavioral activity starts later in the night (A) while under lengthening T-cycles, behavior is increasingly confined to the first half of the night (B). C. The average running wheel activity distribution (+/− SEM, shaded area) during a T-cycle duration of 22 hours (T = 22 h, N = 14 animals) or 26 hours (T = 26 h, N = 14 animals). The dark bar above the behavioral activity indicates the night. D. During short T-cycle durations, the median time of behavioral activity is significantly later in the night than under long T-cycles (***P<0.001, **P<0.01). E. The fraction of behavioral activity in the first versus the second half of the dark period. Behavioral activity shifts to the second half of the dark period during short T-cycles, and to the first half of the dark period during long T-cycles (***P<0.001, T = 22 h compared to all other cycle durations, **P<0.01, *P<0.05, T = 26 h compared to T = 23 h and T = 24 h).

To investigate whether the observed shift in time of the trough of the SCN electrical activity rhythm led to similar displacement of the behavioral activity pattern, we recorded home cage wheel running activity under incrementally shortened or lengthened T-cycles. During a 24 hour T-cycle, animals ran in their wheel from the beginning of the dark period onwards, with most of the intense wheel running during the first part of the dark period (Figs. 2A and B). When T-cycles were gradually shortened, behavioral activity delayed towards a later point in the night (Fig. 2A and C). The median of the behavioral activity distribution shifted from 3.74 h+/−0.16 (T = 24 h) to 6.03 h+/−0.37 (T = 22 h) hours after the onset of darkness (Fig. 2D). Furthermore, most of the wheel running occurred during the second half of the dark period (Fig. 2E). When T-cycles were lengthened, the median of behavioral activity advanced towards the start of the dark period (from 3.74 h+/−0.16 in T = 24 h to 2.94 h+/−0.13 in T = 26 h). The fraction of behavioral activity in the second half of the dark period decreased significantly (P<0.01, Fig. 2E).

### Behavioral Activity Matches the Trough in the SCN Electrical Activity Waveform

**Figure 3:**
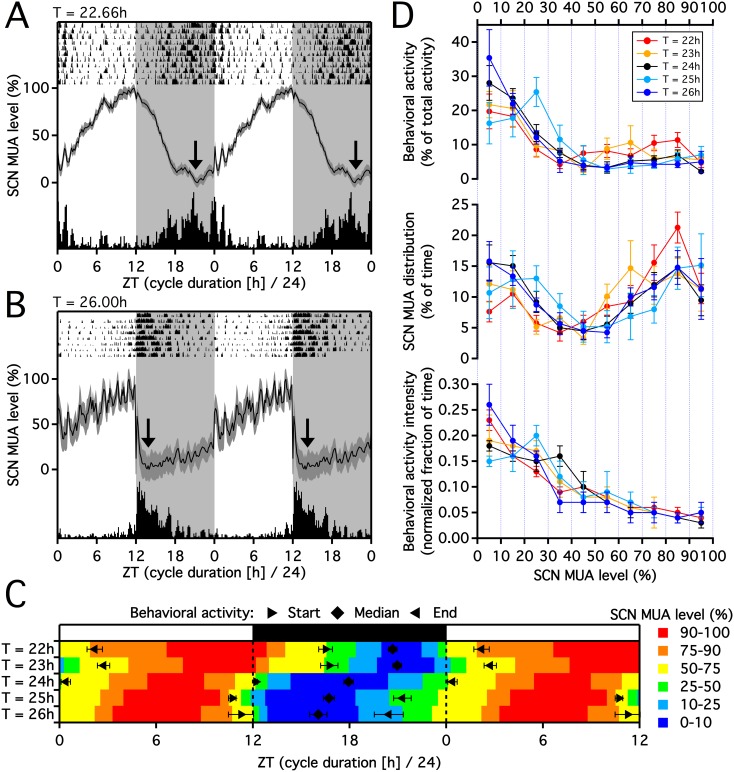
Relation between behavioral activity and SCN electrical impulse frequency. A, B. The average SCN electrical activity waveform and behavioral activity pattern of an individual animal during 11 days in a 22.66 hour T-cycle (A) and during 8 days in a 26 hour T-cycle (B). SCN electrical activity (MUA) levels were normalized to trough (0%) and peak (100%) levels (black line; +/− SEM, grey band). Data are double plotted and dark period is indicated by a grey background. Actograms of locomotor activity are plotted above the averaged electrical activity waveform and the averaged behavioral activity profile is plotted below it. C. During all T-cycle durations, the median of the behavioral activity occurred when the SCN electrical activity level was between 0% and 10% (blue). The start of the active phase occurred around half-maximum SCN electrical activity level for all T-cycles. D. The relationship between behavioral activity and SCN electrical activity levels during different T-cycle durations. Data were pooled in 10% electrical activity bins between trough and peak. The top panel shows the percentage of total daily behavioral activity as a function of SCN electrical activity. The middle panel shows the fraction of the T-cycle during which electrical activity was at each level. The bimodal distribution shows that SCN electrical activity is at low or high levels for a larger fraction of the T-cycle than for intermediate levels. The bottom panel shows the intensity of behavioral activity for each 10% SCN electrical activity level bin. The intensity was largest between 0% and 10% SCN electrical activity level in all T-cycle durations except T = 25 h. Cycle length had no significant effect on the relationship between SCN electrical activity level and the intensity of behavioral activity (*P*>0.05).

Simultaneous recordings of behavioral activity and SCN electrical impulse frequency revealed a close match between these two variables under all T-cycle durations (Fig. 3A and B). A comparison of the timing of the SCN electrical activity trough and the timing of the behavioral activity under different T-cycle durations revealed similar changes in both parameters (Fig. 3C). The median of the behavioral activity time differed significantly between the various T-cycle durations (Table 1). The median behavioral activity time was not different from the time of the SCN electrical activity trough (P>0.01, Bonferroni corrected paired samples t-tests). For all T-cycle durations, the start of the active phase occurred within one hour of the half-maximum level on the declining slope of SCN electrical activity. The onset of behavioral activity was in the middle of the dark period in T = 22 h and T = 23 h, at the start of the dark period in T = 24 h, and slightly before the start of the dark period in T = 25 h and T = 26 h (Fig. 3C). The end of the active phase coincided with the half-maximum SCN electrical activity level on the rising slope in T = 24 h. In T = 22 h and T = 23 h, behavioral activity ended during the first hours of the light period, when SCN electrical activity levels were above half-maximum. In T = 25 h and T = 26 h, behavioral activity ended before the start of the light period, when SCN electrical activity levels were below half-maximum (Fig. 3C).

### SCN Electrical Activity Levels are Inversely Related to the Intensity of Behavioral Activity

We determined the relationship between SCN electrical activity and the level of behavioral activity, and found that the largest fraction of behavioral activity occurred between 0 and 10% of SCN electrical activity amplitude for all T-cycle durations except T = 25 h. SCN electrical activity coded for the level of behavioral activity when the amplitude of electrical activity was below 50%. However, when SCN electrical activity was above half-maximum values, behavioral activity was at a constant low level. This was true for all T-cycle durations (Fig. 3D, top panel). The histogram of SCN electrical activity level as a fraction of time revealed a bimodal distribution (Fig. 3D, middle panel). In this bimodal distribution, the SCN electrical activity was at high levels (70–100%) or low levels (0–30%) for a larger fraction of the T-cycle than at intermediate electrical activity levels (30–70%). A quantification of the intensity of behavioral activity as a function of SCN electrical activity level revealed that behavioral activity was most intense at the trough of SCN electrical activity and decreased with increasing SCN electrical activity (Fig. 3D, bottom panel). We used linear regression analysis to quantify the slope of the decline for each T-cycle duration and found that the relationship between behavioral intensity and SCN electrical activity was not affected by T-cycle duration (*P* = 0.47, F-Test comparing the slope of the regression lines).

### Suppressing SCN Electrical Activity Levels Stimulates Behavioral Activity

**Figure 4:**
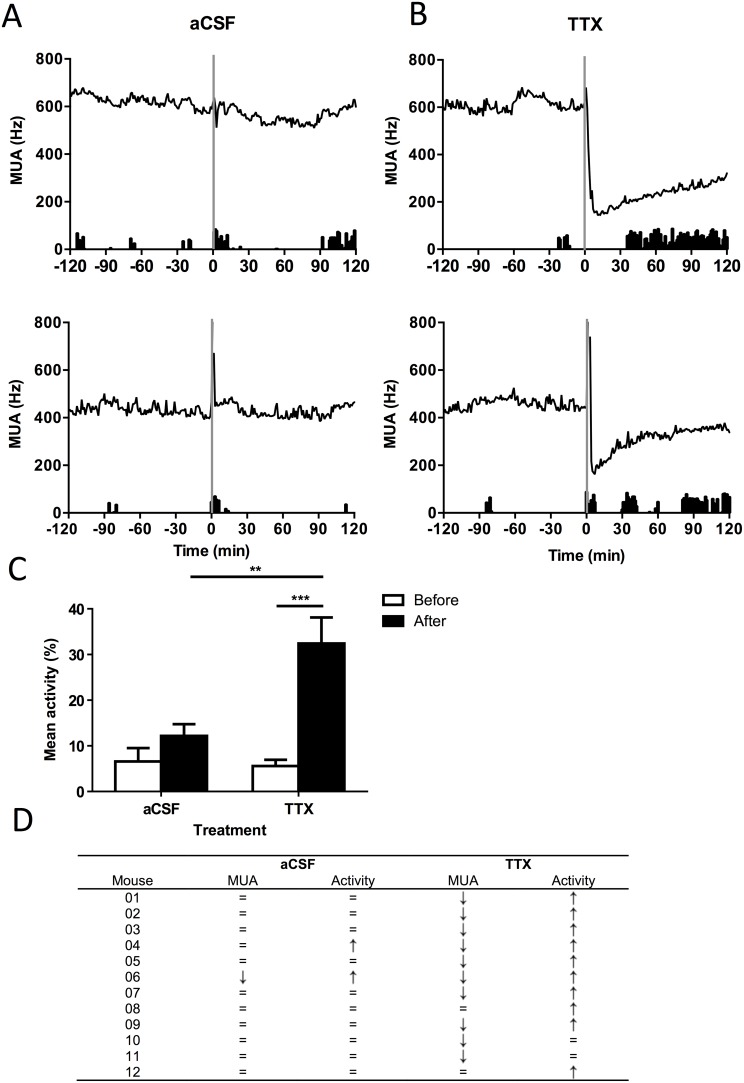
Suppressing SCN electrical activity levels stimulates behavioral activity. A. Exemplary data from 2 individual animals injected with aCSF into the SCN. Mice were injected in constant darkness at CT6 (t = 0). The behavioral activity profile is plotted below the SCN electrical activity (MUA) levels for two hours before and two hours after the injection. B. Exemplary data from 2 individual animals injected with TTX into the SCN. C. The mean behavioral activity two hours preceding and two hours following the aCSF or TTX injection. D. The relationship between behavioral activity and SCN electrical activity following the SCN injections. There were some interindividual differences observed; 2 animals did not respond with a suppression to TTX, and 2 animals did not respond with an increment in activity. In total, twelve mice were injected with TTX and aCSF (randomized; injections at least four days apart). Suppressing SCN electrical activity using TTX significantly increased behavioral activity (P<0.01).

We determined the direct effect of SCN electrical activity levels on behavioral activity by injecting TTX or vehicle (aCSF) as control directly into the SCN while recording the electrical activity of the SCN. The animals received the injection at CT 6, which is the time of the peak in electrical activity, and also the time that behavioral activity is low. In the two hours preceding the injection of either vehicle or TTX, the behavioral activity of the mice was not different. Vehicle injection into the SCN via the guide cannula did not affect SCN electrical activity (Fig. 4A). Following the injecting, the animals displayed a small and brief increase in behavioral activity, which ceased within 10–15 minutes. Injecting TTX into the SCN immediately inhibited SCN electrical activity (Fig. 4B). This suppression in SCN electrical activity coincided with increased behavioral activity as compared to the vehicle injection (*P*<0.01, Fig. 4C). A mixed model analysis, including activity two hours preceding the injections as a covariate, revealed a significant difference in behavioral activity following TTX injection compared to aCSF injection. Responses in electrical and behavioral activity to TTX and vehicle injections are indicated per animal (Fig. 4D).

## Discussion

Our results show that exposing animals to T-cycles with durations of 22–26 h led to reproducible changes in the SCN electrical activity waveform. For all T-cycles, the intensity of locomotor behavior appeared inversely related to the level of SCN electrical activity. The negative correlation between SCN electrical activity and behavior was present not only at circadian timescale, but also within the active phase of the animal. Small changes in T-cycle duration induced large timing differences in the trough and peak of SCN electrical activity, and concomitantly, in the timing of the most intense behavioral activity. The data favor the interpretation that the SCN electrical activity level carries information that determines the temporal profile of behavioral activity, even at a small timescale. This interpretation was supported by experiments showing that acute suppression of electrical activity led to acute increments in behavioral activity.

### Relationship between SCN Electrical Activity Waveform and Behavior

Changes in T-cycle duration had strong effects on the waveform of the SCN electrical activity rhythm. The effect of T-cycle duration was most evident for the trough of the SCN electrical activity, which was in the first half of the night in long T-cycles and in the second half of the night in short T-cycles. The influence of T-cycle duration on SCN waveform is different from the seasonal effects on waveform. Seasonal adjustment to photoperiod changes involve primarily an adaptation of the peak width, i.e. of the daily duration of enhanced SCN activity, while peak and trough time fall in the middle of the day and night [20], [21]. This differs from the distinct sawtooth waveforms induced by long and short T-cycles, which result in quite different peak and trough times.

We observed a consistent relationship between the length of the external T-cycle and the timing of the behavioral activity profile, in line with earlier behavioral studies [22]–[24]. When the T-cycle duration was equal or longer than 24 h (T≥24 h), behavioral activity started at the onset of the dark period and was concentrated in the first hours of the night (Figs. 2B, C and E). When T-cycles were shorter than 24 h (T<24 h), the animal’s active phase started later in the dark period and behavioral activity levels peaked later in the night (Figs. 2A, C and E). The endogenous period of C57Bl/6J mice is about 23.5–23.7 h [17], [25], [26]. Thus, T-cycle durations of 24 h and longer represent the situation where the exogenous period is longer than the endogenous period, whereas T-cycles of 22–23 h are shorter than the endogenous period. The results indicate that changes in T-cycle duration close to the endogenous period are critical to the waveform of the SCN.

Analysis of the relationship between SCN impulse frequency and behavioral recordings revealed that under all T-cycle durations, the decline below half-maximum impulse frequency levels coincided with the onset of behavioral activity. Furthermore, within the animals’ active phase, the intensity of behavioral activity showed a strong inverse relationship with SCN electrical activity levels, which was unaffected by T-cycle duration (Figs. 3A and B). Thus, behavioral activity remained anchored to the trough in electrical activity. Together, our data strongly suggest that the adjustments of behavioral patterns to short and long T-cycles can be attributed to waveform changes of the SCN electrical activity rhythm.

### SCN Electrical Activity Encodes the Pattern of Behavioral Activity

Our analyses revealed a strong negative correlation between the intensity of behavioral activity and SCN electrical activity levels at an ultradian timescale. This relation was present under all T-cycles. Behavioral activity was maximal when SCN firing rate was at trough levels and increase of SCN electrical activity above trough level coincided with a linear decrease in the intensity of behavioral activity up to half maximum SCN electrical activity levels. Between half-maximum and peak firing rate of the SCN, behavioral activity was suppressed to a constant low level (Fig. 3D). Previous studies indicated that SCN electrical activity determines whether the animal is in the active or resting phase [13]. The current results provide first evidence that the level of SCN impulse frequency may in fact be actively involved in regulating the driving force for behavioral activity within those temporal windows. This hypothesis was verified in an additional experiment in which we used TTX to attenuate SCN electrical activity levels, to levels below half-maximum levels. The combination with the recording electrodes allowed us to monitor resulting changes in SCN impulse frequency to verify the induction of physiological changes in neuronal activity.

The implantable electrode fused with infusion cannula were aimed at the mid of the SCN, but our histology indicates that they are often located more laterally. Even though it is likely that TTX may not have affected the entire SCN, we have lowered the electrical activity of the SCN as a whole, by acute suppression of at least part of the SCN. We conclude that the overall suppression caused an immediate increase in locomotor activity, but do not expect we have suppressed the activity of all SCN neurons.

The SCN project monosynaptically to the sub-paraventricular zone (SPVZ) which oscillates in antiphase with the SCN [27]. The SPVZ is located dorsal to the SCN and is a major output pathway [28]. Enhanced activity of the SPVZ is associated with increments in behavioral activity [27], [29]. It is possible that the TTX-induced decrease in SCN activity leads to immediate activation of the SPVZ, thereby inducing behavioral activity.

Our results are in agreement with Hu *et al*. [30], [31], who observed that following SCN lesions, scale invariant patterns in locomotor activity was changed over a broad range of time scales, from minutes to over 24 h. The authors suggested that the SCN does not act only as a generator of circadian oscillations. Our data are in full support of their interpretation and provide direct evidence for a role of the SCN in the temporal organization of behavior, outside the circadian range.

While decreasing levels of SCN activity trigger the increase of behavioral activity levels, it has also been shown that behavioral activity in turn suppresses the activity of the SCN [32]–[34]. This reciprocal influence indicates that these processes are intrinsically intertwined, which changes our view on the organization of the circadian system. That is, the SCN and behavioral activity form a positive feedback loop in which they strengthen each other’s effect.

Rhythmic clock gene expression is required for the presence of neural activity rhythms [35] and, reciprocally, manipulations of membrane electrophysiological function can impact intracellular molecular rhythms [36], [37]. With regard to the tight coupling between SCN neural activity and behavioral activity levels shown in our data, it will be interesting to see whether this extends to the molecular level. Given that circadian waveform changes seen with photoperiod are present both in clock gene expression patterns as well as in the SCN neural activity waveform it may perhaps be expected that the influence of T-cycles on the waveform of circadian neural activity waveform is similarly reflected in the clock gene expression profiles at a circadian timescale. However, the timeframe in which we see changes in behavioral and neuronal activity are in the order of magnitude of seconds to minutes as shown in our TTX experiment. It is unlikely that gene expression profiles operate at this timescale to control behavioral activity changes.

### Chronotypes

Differences in the ratio between the human endogenous period and the environmental (24 h) LD cycle result in differences in timing of behavioral and physiological rhythms relative to the LD cycle [38], [39]. Our results suggest that the ratio between the endogenous cycle length and the LD cycle has consequences not only for onset and offset of behavioral activity, but also for the distribution of behavioral activity between these two time points. We observed strong differences in the timing of behavioral activity and SCN electrical activity between short T-cycle durations (T = 22 h and T = 23 h) and T-cycle duration of 24 hours or longer. Specifically, the shift between early and late behavioral activity largely occurred within the narrow boundary between T = 24 h and T = 23 h, where a 1 hour difference in cycle duration corresponded to a 3.5 hour difference in behavioral activity relative to the T-cycle. Interestingly, this phase-jump coincides with the border between T-cycles that are longer and shorter than the endogenous period of mice in constant darkness (23.5–23.7 h [17], [25], [26]). This observation matches the finding in mice with clock-gene mutations [40] as well as in humans [41] that small differences in the endogenous circadian period can underlie a large difference in chronotype [42]. In humans, the mean endogenous circadian period is thought to be slightly over 24 hours with a narrow distribution ranging from 23.75 up to 25 hours [43]–[45]. This relatively narrow distribution in endogenous period leads to a much broader distribution in the phase of entrainment, with core body temperature trough and mid-sleep time both ranging fKrom 2 am to 9 am [43], [46]. Thus, small differences between internal and external cycle time can induce waveform changes of the SCN rhythm, that can consequently lead to large differences in the preferred timing of behavior of different chronotypes.
